# Supplementary material for: A prospective observational study on persistent postoperative pediatric pain, 4P: The study protocol
Source: PLoS One. 2025 Jan 14;20(1):e0316533. doi: 10.1371/journal.pone.0316533 (PMC11731717; doi:10.1371/journal.pone.0316533)
Supplement: Attachment — Submitted filename: Response to Reviewers.docx [file pone.0316533.s001.docx]

Dear Academic Editor and Reviewers, December 10 2024

Thank you for considering this protocol for publication and for your comments. Below you can find our responses to comments from the editor and reviewer:

Journal Requirements:

When submitting your revision, we need you to address these additional requirements.

1. Please ensure that your manuscript meets PLOS ONE's style requirements, including those for file naming. The PLOS ONE style templates can be found at

<https://journals.plos.org/plosone/s/file?id=wjVg/PLOSOne_formatting_sample_main_body.pdf> and 
<https://journals.plos.org/plosone/s/file?id=ba62/PLOSOne_formatting_sample_title_authors_affiliations.pdf>

Author's comment: We have adapted the manuscript according to the instructions.

2. We note that the grant information you provided in the ‘Funding Information’ and ‘Financial Disclosure’ sections do not match.

When you resubmit, please ensure that you provide the correct grant numbers for the awards you received for your study in the ‘Funding Information’ section.

Author´s comment: The grants we have received do not have grant numbers. We have corrected the information given under “funding information” and “financial disclosure” and deleted the information on funding from the manuscript file.

3. Please note that funding information should not appear in the Acknowledgments section or other areas of your manuscript. We will only publish funding information present in the Funding Statement section of the online submission form. Please remove any funding-related text from the manuscript.

Author´s comment: Thank you, it has now been removed from the manuscript.

4. Please include captions for your Supporting Information files at the end of your manuscript, and update any in-text citations to match accordingly. Please see our Supporting Information guidelines for more information: <http://journals.plos.org/plosone/s/supporting-information>.

Author´s comment: We have provided captions at the end of the manuscript accordingly.

5. Please review your reference list to ensure that it is complete and correct. If you have cited papers that have been retracted, please include the rationale for doing so in the manuscript text, or remove these references and replace them with relevant current references. Any changes to the reference list should be mentioned in the rebuttal letter that accompanies your revised manuscript. If you need to cite a retracted article, indicate the article’s retracted status in the References list and also include a citation and full reference for the retraction notice.

Author´s comment: We cannot find any retracted manuscripts. Please be more specific if there is something we have missed.

Reviewers' comments:

Reviewer's Responses to Questions

**Comments to the Author**

1. Does the manuscript provide a valid rationale for the proposed study, with clearly identified and justified research questions?

The research question outlined is expected to address a valid academic problem or topic and contribute to the base of knowledge in the field.

Reviewer #1: Yes

2. Is the protocol technically sound and planned in a manner that will lead to a meaningful outcome and allow testing the stated hypotheses?

The manuscript should describe the methods in sufficient detail to prevent undisclosed flexibility in the experimental procedure or analysis pipeline, including sufficient outcome-neutral conditions (e.g. necessary controls, absence of floor or ceiling effects) to test the proposed hypotheses and a statistical power analysis where applicable. As there may be aspects of the methodology and analysis which can only be refined once the work is undertaken, authors should outline potential assumptions and explicitly describe what aspects of the proposed analyses, if any, are exploratory.

Reviewer #1: Yes

3. Is the methodology feasible and described in sufficient detail to allow the work to be replicable?


Descriptions of methods and materials in the protocol should be reported in sufficient detail for another researcher to reproduce all experiments and analyses. The protocol should describe the appropriate controls, sample size calculations, and replication needed to ensure that the data are robust and reproducible.

Reviewer #1: Yes

4. Have the authors described where all data underlying the findings will be made available when the study is complete?

The [PLOS Data policy](https://track.editorialmanager.com/CL0/https:%2F%2Fjournals.plos.org%2Fplosone%2Fs%2Fmaterials-and-software-sharing/1/010f019356149c83-a4f4d7a2-4495-4153-8ad3-113ef49be60a-000000/l_ABcIZ36fcy8-G02mTPogT1UC66YiFJ5XLhOFp4IqM=186) requires authors to make all data underlying the findings described in their manuscript fully available without restriction, with rare exception, at the time of publication. The data should be provided as part of the manuscript or its supporting information, or deposited to a public repository. For example, in addition to summary statistics, the data points behind means, medians and variance measures should be available. If there are restrictions on publicly sharing data—e.g. participant privacy or use of data from a third party—those must be specified.

Reviewer #1: Yes

5. Is the manuscript presented in an intelligible fashion and written in standard English?

PLOS ONE does not copyedit accepted manuscripts, so the language in submitted articles must be clear, correct, and unambiguous. Any typographical or grammatical errors should be corrected at revision, so please note any specific errors here.

Reviewer #1: Yes

6. Review Comments to the Author

Please use the space provided to explain your answers to the questions above and, if applicable, provide comments about issues authors must address before this protocol can be accepted for publication. You may also include additional comments for the author, including concerns about research or publication ethics.

You may also provide optional suggestions and comments to authors that they might find helpful in planning their study.

Reviewer #1: Although the study has been performed before numerous times, I salute you for tackling this important subject.
I would add that many variables should taken into consideration in your statistical model as surgery time, surgical complications, patient comorbidities and psychological evaluation of the child before surgery

Author’s comment: Thank you for your important comments.

We are aware of the fact that several variables need to be taken into consideration.

*Surgery time, surgical complications*: we have not included this in the registration and these important issues need to be addressed in the discussion section of future publications. We have grouped our types of surgery into groups of similar surgery types as well as expected similar surgery times. It would have been of interest to know the exact surgery time and we will address this in discussions of future publications.

*Patient comorbidities and psychological evaluation before surgery*: We do not register comorbidities; however, we register both pain and psychological well-being before surgery.

7. **Do you want your identity to be public for this peer review?**

Reviewer #1: **Yes:** Mahmoud Elfiky

While revising your submission, please upload your figure files to the Preflight Analysis and Conversion Engine (PACE) digital diagnostic tool, [https://pacev2.apexcovantage.com/](https://track.editorialmanager.com/CL0/https:%2F%2Fpacev2.apexcovantage.com%2F/1/010f019356149c83-a4f4d7a2-4495-4153-8ad3-113ef49be60a-000000/HaercbI6QRv0rdZV6AM1B1D99mlWfqBdDxhwdqwciLc=186).

Author’s comment: Figure 1 and 2 have been uploaded in PACE. The revised version will be added in the revised version of the submission.
